# Incidence and costs of bleeding-related complications in French hospitals following surgery for various diagnoses

**DOI:** 10.1186/1472-6963-13-186

**Published:** 2013-05-22

**Authors:** Xin Ye, Antoine Lafuma, Elodie Torreton, Axel Arnaud

**Affiliations:** 1 Ethicon, Inc., Room D207, Route 22, West Somerville, NJ, USA; 2 Cemka-Eval, 43 boulevard du Maréchal Joffre, Bourg la Reine, France; 3 Ethicon SAS, 1 rue Camille Desmoulins, Issy les Moulineaux, France

**Keywords:** Bleeding, Surgery, France, Costs, Incidence rate

## Abstract

**Background:**

Limited information is available on the epidemiology and economics of bleeding during surgery in France.

**Method:**

The objective of this study was to examine the incidence, costs and length of stay (LOS) of bleeding-related complications during various surgical procedures.

A retrospective DRG (diagnosis-related group) analysis was conducted using the French National database PMSI (Programme Médicalisé des Systèmes d’Informations). Patients undergoing surgery during 2008 were identified according to their DRG classifications and those with at least one episode of secondary haemostasis and blood transfusion (according to French procedure codes) were designated as ‘with bleeding’ (WB). The analysis focussed on DRGs where ≥10% of patients presenting with bleeding and compared them to patients who did not require blood transfusions (i.e. without bleeding: WoB).

Hospital LOS and costs (age and gender adjusted) were compared between WB and WoB patients within each DRG. Hospitalisation periods exceeding the LOS average (derived from the PMSI database) were compared using multivariate logistic regression adjusted for patient characteristics.

**Results:**

Amongst all 88 different surgical DRGs recognised by the French database 24 (totalling 321,657 hospitalisations) yielded WB rates ≥10% (range 10.3–25.3%). The highest DRG rates were for transplantations, cardiac and major orthopaedic surgery, vascular and solid organ surgery.

The average LOS for the WB population (adjusted for age, gender, number of procedures and secondary diagnoses) were 3.38 days (26.5%) longer than for the WoB population (p < 0.001). The average adjusted LOS costs for a WB patient were estimated at €12,087, and for a WoB patient €10,086 i.e. 19.9% higher than for WB patients (p < 0.001). The overall mean LOS was exceeded by 42.3% of WB patients compared to 37.0% of WoB patients (p < 0.001). Also, WB patients were more likely to exceed the average LOS than were WoB patients (Odds Ratio (OR) = 1.09, p < 0.001), after adjusting for patient characteristics.

**Conclusions:**

The present study for France demonstrates a significant increase of hospital LOS and associated costs following post-surgical bleeding, supporting the need for blood conservation strategies.

## Background

Bleeding is a frequent complication following surgery that can lead to substantial morbidity and mortality [1,2]. During surgery, or when managing trauma, surgeons may be faced by mild to severe bleeding necessitating simple or intricate procedures according to anatomical location, the proximity of adjacent structures, and type of tissue harbouring the bleeding source. In such situations surgeons must decide how best to stem the bleeding using available products and techniques together or sequentially.

Insufficient haemostasis can increase operative time and when excessive may need whole blood or blood constituent transfusions. Further complications include longer hospitalisation, risk of infection, and ventilation requirement. Haemostasis is usually performed mechanically (sutures, ligatures, clips, staples) or with various electrically generated coagulation systems (electrocautery, argon beam lasers, ultrasound) [3,4].

Blood transfusions and reoperations are not uncommon sequellae when excessive bleeding follows surgery, even in France where blood transfusions have dramatically decreased because of AIDS and hepatitis risks [5,6]. Between 1992 and 2001 the French Blood Public Organisation [7] reported a fall of 2 million (23.6%) blood transfusion units administered per year. In 2009 the National Blood Bank delivered a total of 2.2 million such units in France, with 25% administered to surgical patients [8] mainly for trauma, orthopaedic surgery and cardiac surgery.

Post-surgical bleeding complications can create a significant economic impact. Boucher and Hannon [9] estimated that haematocyte transfusions in the US cost from $1,840 to $2,760 per unit, plus operating theatre time of $1,890 to $3,150, intensive care unit supervision, and ventilator support, amounting in total to $4,810.

Little information is currently available on bleeding complication rates and related costs in European countries. Hence the availability of a fairly comprehensive hospital database in France provides a unique opportunity to examine this important issue. For the present study we assumed that bleeding episodes could be identified in the database. Such information would permit a comparison of hospitalisation costs both with and without blood loss.

The primary objectives of the present French hospital database analysis were: (1) to estimate the frequency of surgical DRGs associated with blood or blood constituent transfusions administered to ≥10% of WB patients; and (2) to compare the estimated costs of bleeding episodes and transfusions required by these patients *versus* the costs of WoB patients drawn from similar surgical DRGs.

## Methods

### French hospital database PMSI-MCO

The French computerised hospital database for medicine, surgery and obstetrics (PMSI-MCO: Programme Médicalisé des Systèmes d’Informations – Médecine Chirurgie Obstétrique) covers all short-term hospitalisations in France (about. 20 million public and private admissions per annum). It comprises a standardised case record compiled at discharge after each short-term hospitalisation and encodes administrative and medical data with standardised classifications and nomenclatures.

The system is derived from the US DRG classification and employs a decision tree that leads each hospitalisation towards its appropriate diagnostic or surgical procedures group (Groupe Homogène de Malades: GHM). Accessible data for all patients include age, gender, admission and discharge addresses, primary and secondary diagnoses, treatment procedures, DRG codes, admission duration, intensive care resources deployed, hospital identification, public or private sector, etc.

Nomenclature adopted the X^th^ revision of the International Classification of Diseases (ICD-10) of the World Health Organization [10], defining conditions by one primary diagnosis and up to 20 secondary diagnoses per hospitalisation, plus the French classification of medical procedures “La Classification Commune des Actes Médicaux” (CCAM), available as a full listing [11]. Thus medico-administrative (PMSI) variables plus one surgical act were sufficient to classify a hospitalisation as a ‘surgical’ DRG. After 2004, global hospitalisation budgets were progressively replaced by new budgets linked to specific activities (T2A Tarification A l’Activité). The new procedure was fully in place by 2008. Official tariffs per DRG in French hospitals are revised and issued every 6 months by the French Ministry of Health [12]. These tariffs per DRG are charged to the Sickness Funds for each hospitalisation. These tariffs were not used for our costing.

### Study patients

The study was limited to surgery and included patients of all ages. DRG classification was performed at hospital discharge in 2008, hence some patients hospitalised in 2007 were included and others not discharged until 2009 were necessarily excluded. All hospitalisations extended to at least 2 nights. Shorter hospitalisations were less documented and no death was recorded in these short stays. Patient inclusion was based on surgical DRGs identified in the French system by a third letter coding ‘C’ for surgery (Chirurgie). A surgical procedure was the primary criterion specifying such DRGs.

Procedures such as aortic aneurysm repair are not classified as surgery.

### Bleeding-related complication and/or blood product transfusion status

Bleeding-related complications and/or blood product transfusions were identified according to diagnosis and procedure codes relating to bleeding complications. Patients were divided into two groups, i.e. with bleeding (WB) or without bleeding (WoB) provided *one* of the following codes was retrieved for the hospitalisation: (1) Procedures codes (i.e. 11 CCAM codes: Additional file 1) for secondary haemostasis following index surgery (not preventative transfusions); or (2) one of the four following ICD Diagnosis codes: T81.0 for haemorrhage or haematoma complicating a procedure; Z513 for blood products transfusion; Y63.0 for natural blood and blood products; and Y446 for transfusion reactions.

It should be noted that no information was available concerning which blood products were transfused, since their costs were not included in the database. As diagnoses and codes for transfusion procedures were confounded, actual transfusion rates may be underestimated by the lack of specific coding. Also, surgical patients identified by bleeding, transfusions, or other haemorrhagic outcomes may have been coded for a manifestation of their underlying condition, or for a complication of surgery, with no possibility of identifying the real cause of bleeding. Hence in this manuscript ‘bleeding’ necessarily confounds bleeding complications and transfusions. Data concerning pre-operative bleeding risks were not available.

### Statistical analysis

Hospitalisations associated with bleeding complications were extracted for each diagnosis-related group and their frequencies were ranked across DRGs. Surgical DRGs presenting where ≥10% of patients presented with bleeding were selected for further analysis. Control patients were those without bleeding (WoB) drawn from similar surgical DRGs. The ≥10% criterion was chosen empirically to include sufficient WB and WoB group sizes for comparisons of costs and LOS outcomes. DRGs were merged when they involved patients within the same organ classification undergoing similar surgical procedures with bleeding differences (<3%) (Additional file 2) to increase the power of the statistical analysis.

Comparisons of WB and WoB groups within each merged DRG were based on patient characteristics (age and gender) and DRG aspects unrelated to the classification, e.g., number of procedures and secondary diagnoses. Patient groups were compared for age, gender, number of procedures, number of diagnoses, adjusted length of stay (LOS) and costs, using non-parametric statistics (Wilcoxon). Cost estimates were derived from the French National Costs Scale based on information from a sample of hospitals using similar accounting methods and linked to the PMSI. This enabled statistical and economic analyses to calculate average costs per DRG. Separate scales were applied to public and private sectors. Cost estimates per DRG comprised total hospital costs including overheads and public sector physicians’ salaries and private sector physicians’ fees not included in the tariffs. The foregoing standard costs were applied to DRGs according to the hospital sector. No difference was observed in the bleeding rates between sectors.

Rates of hospitalisations for WB and WoB surgery exceeding the PMSI average LOS were compared using multivariate logistic regression that included confounding variables. The analysis was kept simple and objective, i.e. bleeding *versus* non-bleeding cases. Confounding variables were selected that primarily permitted comparisons of DRGs (with and without bleeding) according to patient characteristics (age and gender), LOS, numbers of diagnoses and procedures. Convergence within the model and statistically significant covariates were checked. No centre effect was included in the analyses as hospitals belonging to regional networks could not be precisely identified in the database.

Prior to comparisons, costs of hospitalisation with and without bleeding were calculated per surgical group and complication status. Adjustments for age, gender, numbers of procedures and secondary diagnoses were performed for the WB complication groups based on the difference observed between the WB group and the WoB group taking the WoB group as the reference. Transfer of patients to other acute care hospitals was rare and can be ignored.

Statistical analyses were performed using SAS Statistical Software #9.2 (SAS Institute Inc., North Carolina, USA) with a 5% significance threshold for all tests.

## Results

### Surgical DRGs with and without bleeding

In total, 88 different surgical DRGs resulting in 1,720,486 hospitalisations in 2008 were identified in the French PMSI database. Hospitalisations with bleeding complications (defined previously) totalled 77,521 cases (4.5%). More than 95% of WB cases were identified by at least one code relating to a transfusion.

**Figure 1:**
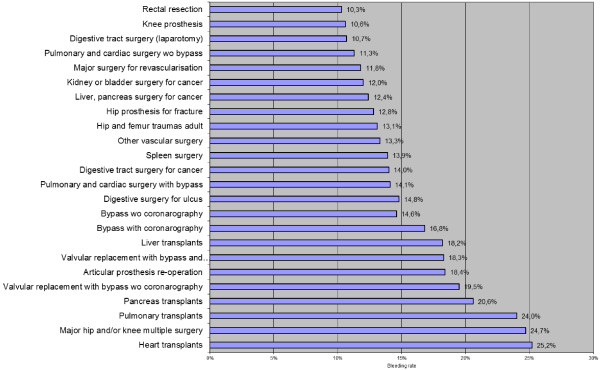
Surgical DRGs (≥10%) ranked by WB rate.

Twenty-four surgical DRGs (totalling 321,657 hospitalisations) were characterised by ≥10% of patients considered as with bleeding (Figure 1).

**Figure 2:**
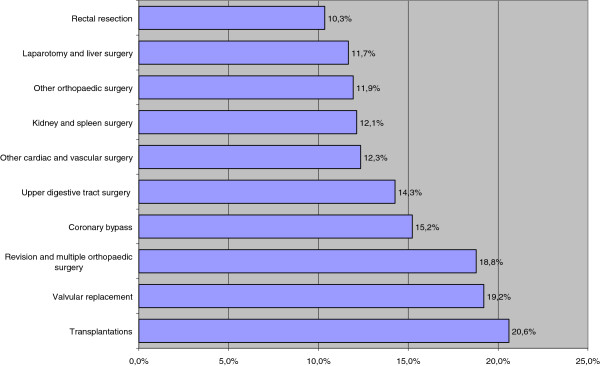
Rate of bleeding related consequences (Top 10 surgical groups).

The latter DRGs were combined when their WB rates and surgical interventions were similar and corresponded to the same organ group. Ten surgical DRGs with the highest proportion of patients given blood or blood constituent transfusions are shown in Figure 2.

### Hospital LOS and costs

**Figure 3:**
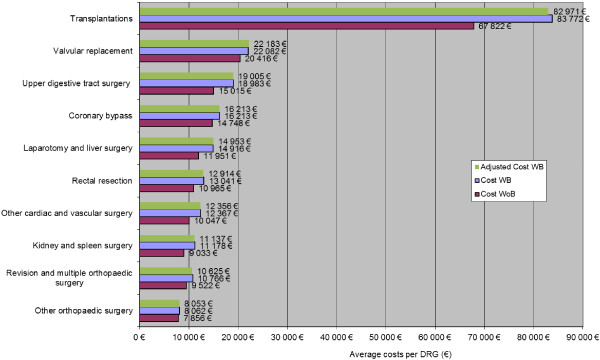
Costs associated with bleeding related consequences (Top 10 surgical groups) with costs adjusted for the WB group.

**Figure 4:**
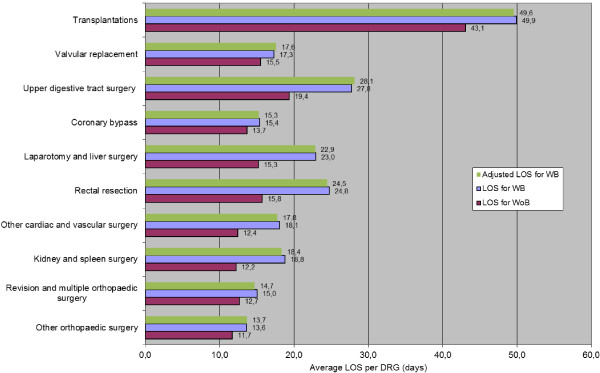
LOS (Days) associated with bleeding related consequences (Top 10 surgical groups) with adjusted LOS for the WB group.

**Figure 5:**
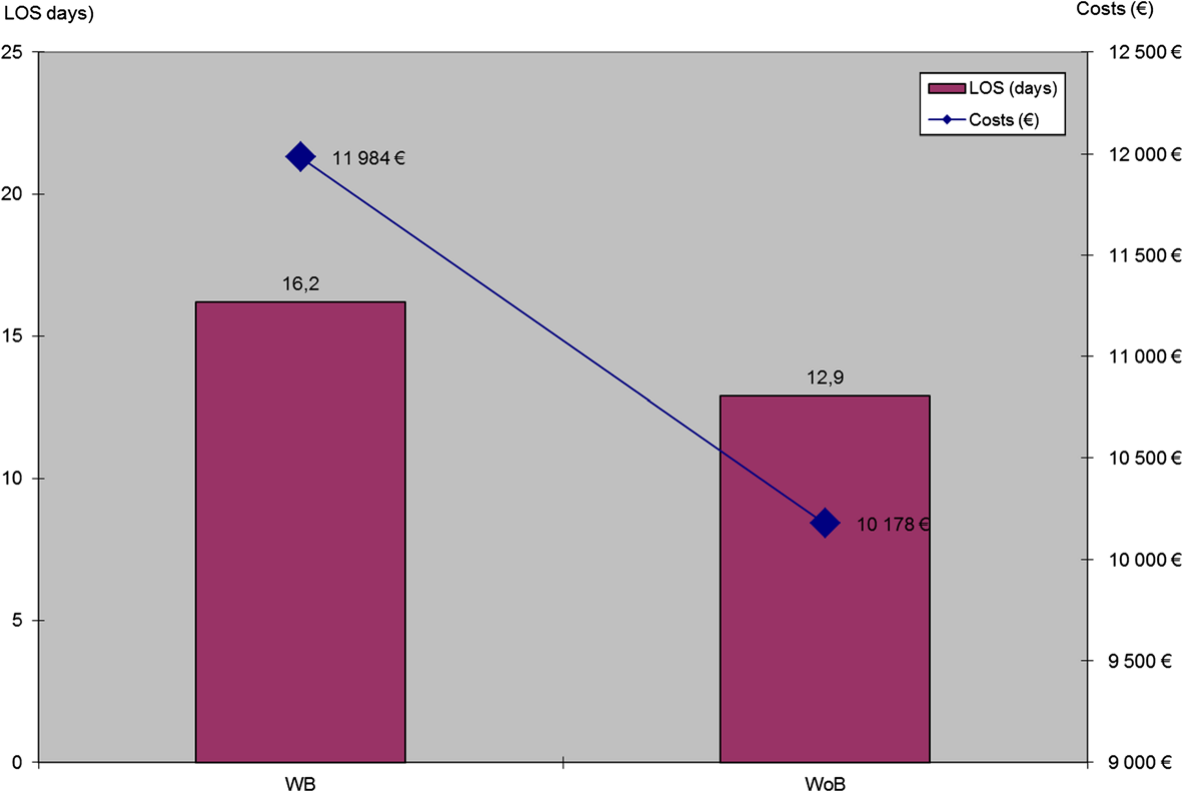
Adjusted mean LOS (p < 0.0001) and Costs (p < 0.0001) according to the WB status.

Hospital LOS and costs of surgical DRGs for WB patients were compared with those of surgical WoB patients. Figures 3 and 4 depict unadjusted total hospitalisation durations (LOS) and costs according to surgical group and complication status. Overall LOS and total hospital costs are displayed across all surgical groups in Figure 5, according to surgical group and complication status, with adjustment for age, gender and number of procedures performed. On average, surgical LOS was longer for WB than WoB patients (16.2 vs. 12.9 days) and their respective costs were higher (€11,984 vs. €10,178) across all DRGs. Also, unadjusted average LOS was longer for WB than WoB patients (16.6 vs. 12.9 days) and respective costs were higher (€12,147 vs. €10,178).

### Rate of stays over the LOS mean

**Figure 6:**
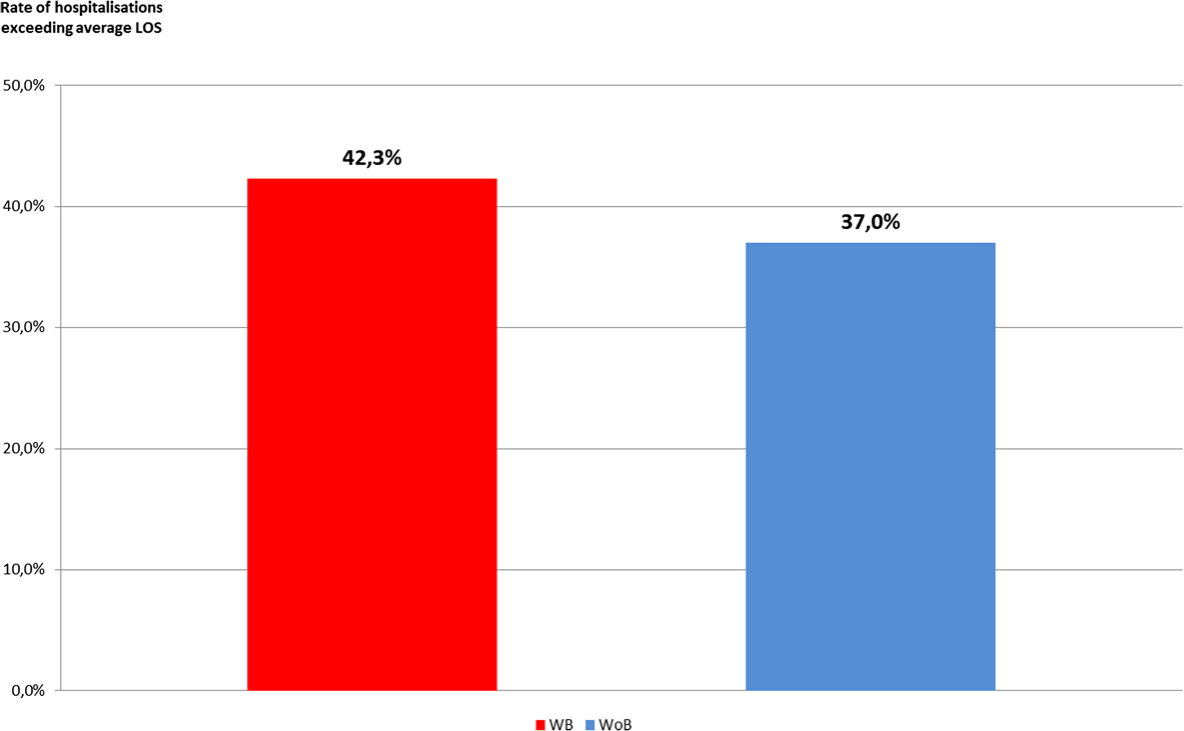
Percentages exceeding average LOS.

Hospitalisations of WB patients were classified as above or below the mean LOS for every surgical DRG associated with severe blood loss. A global analysis was then performed of hospitalisation durations above the LOS mean, comparing percentages of WB and WoB patients across all DRGs. The results are shown in Figure 6.

A global statistical analysis was performed by logistic regression adjusted for patients’ characteristics. The model included 321,657 observations with 121,147 LOS durations exceeding the overall hospitalisation mean. Five characteristics were included in the model, i.e. patients’ age (<65 to >80), gender, number of surgical procedures (<6, 6–12, >12), number of secondary diagnoses (<3, 3–5, >6) and bleeding (Yes/No, i.e. WB or WoB). Characteristics’ classes were defined empirically to have sufficient sample sizes in each group of each DRG. The model (Table 1) was convergent and all tested variable were included.

**Table 1:** Odd ratios estimates of the logistic regression model

**Effect**		**Point estimate**	**95% confidence interval de Wald**	
Gender	Female vs Male	1.137	1.120	1.155
Age class	>80 years vs <65 years	1.189	1.166	1.213
	65–70 vs <65 years	1.234	1.202	1.266
	>70–75 vs <65 years	1.249	1.220	1.279
	>75–80 vs <65 years	1.394	1.363	1.426
Bleeding	Yes vs No	1.060	1.037	1.083
Number of procedures	>12 procedures vs <6 procedures	1.966	1.926	2.007
	6–12 vs <6 procedures	1.234	1.210	1.259
Number of secondary diagnoses (DAS)	>6 DAS vs <3 DAS	1.129	1.107	1.152
	3–6 DAS vs <3 DAS	1.001	0.984	1.019

The logistic regression global analysis showed that surgical hospitalisations of WB patients posed a significantly higher risk (p < 0.001) of exceeding average hospital LOS durations, compared to cases with no transfusion (OR: 1.09; 95% CI: 1.07-1.11). In addition, other independent variables were also significant. For example, hospitalisations involving >12 surgical procedures were associated with an approximately ×2 risk of exceeding the average LOS, compared to <6 procedures.

## Discussion

Our results indicate that bleeding events during major surgery in France (2008) requiring blood or constituent transfusions were relatively common, although frequencies varied between DRG classes. The three DRG combinations most frequently associated with blood transfusions were heart transplants (25.2%), major hip/knee and multiple orthopaedic interventions (24.7%), and pulmonary transplants (24.0%).

In general, surgical patients requiring blood transfusions were more frequently female, elderly and liable to more secondary diagnoses. Also DRGs involving numerous surgical procedures were more often associated with blood transfusions requirements. After adjustment for age, gender, number of procedures and secondary diagnoses, longer hospital admissions and higher costs characterised surgical DRGs, with ≥10% of patients given blood or blood constituent transfusions. When assessing the LOS durations of WB patients we were obliged to perform indirect analyses based on LOS frequencies in excess of DRG averages, so as to accommodate the French algorithm determining DRG codes. Hospital LOS is an important factor when determining DRG codes. The presence of bleeding complications can lead to more severe and more costly DRGs on account of secondary diagnoses and longer LOS durations. Although longer hospitalisations automatically lead to more severe DRGs, this does not imply a direct linkage between hospital LOS and surgical procedure costs, with or without bleeding complications. Indeed, a rapid and fatal complication could direct a patient’s diagnosis to a less severe DRG because the LOS was insufficiently long.

Very few papers have explored the clinical and economic consequences of severe bleeding during surgery. Most have focussed on how to reduce the need for blood transfusion by screening for anaemia and treating affected patients with erythropoietin stimulating agents, as before elective orthopaedic surgery. We identified a recent US analysis by Morton et al. [13] which estimated the increase in average LOS at 2.5 days and found that costs were $17 194 higher for a transfused cohort compared to controls (p < 0.0001). However, there are many reasons for caution, e.g., transfusion data in the US study may have been more complete than reported here for France, i.e. the US difference was based on all hospitalisations, instead of selected hospitalisations in our study which used a different design; differences between the US and French Healthcare systems also exist in terms of medical practice, hospitalisation durations, and costs. Nonetheless, both studies point to similar conclusions in terms of higher costs associated with bleeding complications.

The present study has several limitations. First, in the absence of a specific directive and hospital record code, whole blood and blood constituent charges are not formally included in the database, hence it is probable that transfusion frequency and costs were under-estimated. This is supported by the following estimate for the 77,521 surgical patients in the present study. A ‘one day’ national survey in 2008 identified 742 surgical patients transfused with a total of 5,765 erythrocyte units, i.e. 7.8 units / patient [8]. Accordingly, the 77,521 surgical patients in the present study would have received a total of 7.8 × 77,521 = 604,663 erythrocyte units. The latter number, however, amounts to just one-third of the 2,013,863 erythrocytes units transfused in French hospitals during 2006 [7]: i.e. 2,013,863 / 604,663 = 3.3.

This bias could be important but should in fact underestimate the difference in costs and LOS as a part of patients with bleeding but not coded so and were included in the WoB group.

Second, because our analysis was retrospective, unreported factors may have confounded relationships between surgical bleeding consequences, LOS, and costs. Third, the PMSI database does not provide cost information, hence costs were estimated from the National costs scales and an application of average costs to surgical DRGs associated with severe blood loss. This limitation did not allow us to take into consideration the specific distributions of costs and LOS.

Fourth, French hospital networks are providing pooled information on their hospitals that does not allow taking into account a centre effect and to study relationship of hospital characteristics and costs with bleeding rates.

Fifth, the methods that we selected could have some impact on our results. Our age groups were large, especially for the younger patients. Even, if paediatric cases were very rare (less than 1%) younger adults could present less frequently bleeding and require less transfusions middle age patients and this could have biased our estimates. Lastly, we did not analyse the influence of the sector on the costs consequences of bleeding because we thought it could be redundant with the patient’s characteristics.

Then, it appears that our study can be considered as a pilot study because of the possible important bias that could have underestimate of overestimate our LOS and costs results. Further and more precise analyses are needed to confirm our results in France as similar studies could also be performed in other countries where DRG systems and costs could be different and lead to other conclusions.

## Conclusion

In conclusion, this study estimated increases of hospital LOS and costs for patients with severe surgical bleeding complications in France. Hospitalisations longer than the average LOS across all DRGs indicated significant costs associated with severe post-surgical bleeding. Awareness of the clinical and economic consequences of severe post-surgical bleeding provides an important framework when evaluating blood conservation strategies.

## Competing interests

Antoine Lafuma (AL) and Elodie Torreton (ET) are employees of Cemka Eval which received funding for the study from Ethicon Inc.

Xin Ye (XY) and Axel Arnaud (AA) were employees of Ethicon Inc. during the study and manuscript preparation.

Ethicon markets a range of products for hemostasis during surgery with standard techniques.

## Authors’ contributions

AL and XY designed the study; AL and ET designed the statistical analysis; AL, AA and XY wrote the manuscript which was reviewed by ET. All authors read and approved the final manuscript.

## Pre-publication history

The pre-publication history for this paper can be accessed here:

http://www.biomedcentral.com/1472-6963/13/186/prepub

## Supplementary Material

Additional file 1 CCAM Procedures codes for secondary haemostasis following index surgery. Click here for file

Additional file 2 **Surgical DRGs identified by >10% of patients receiving blood or blood constituent transfusions.** DRGs were combined when they involved similar surgery with similar bleeding frequencies (differences <3%). Click here for file
