# Supplementary material for: Incidence and costs of bleeding-related complications in French hospitals following surgery for various diagnoses
Source: BMC Health Serv Res. 2013 May 22;13:186. doi: 10.1186/1472-6963-13-186 (PMC3668216; doi:10.1186/1472-6963-13-186)
Supplement: Additional file 1 — CCAM Procedures codes for secondary haemostasis following index surgery. [file 1472-6963-13-186-S1.doc]

Appendix I: CCAM Procedures codes for secondary haemostasis following index surgery.

| Code | Description |
| --- | --- |
| CASA001 | Secondary haemostasis following a surgical act on the ear |
| EBSA012 | Secondary haemostasis following a surgical act on cervical vessels |
| DGSA005 | Secondary haemostasis following a surgical act on the aorta by laparotomy |
| EZSA004 | Secondary haemostasis following a surgical act on limb vessels |
| DZSA002 | Secondary haemostasis following a surgical act on the heart or thoracic vessels by thoracotomy |
| FASD001 | Secondary haemostasis following a surgical act for tonsillectomy or adenoidectomy |
| EBSA011 | Secondary haemostasis following cervicotomy |
| ZBSA001 | Secondary haemostasis following thoracotomy |
| HASD003 | Secondary haemostasis following mouth electrocoagulation |
| HJSD001 | Secondary haemostasis following a surgical act on the rectum |
| HKSD001 | Secondary haemostasis following a surgical act on the anus |
