# Supplementary material for: Incidence and costs of bleeding-related complications in French hospitals following surgery for various diagnoses
Source: BMC Health Serv Res. 2013 May 22;13:186. doi: 10.1186/1472-6963-13-186 (PMC3668216; doi:10.1186/1472-6963-13-186)
Supplement: Additional file 2 — Surgical DRGs identified by >10% of patients receiving blood or blood constituent transfusions. DRGs were combined when they involved similar surgery with similar bleeding frequencies (differences <3%). [file 1472-6963-13-186-S2.doc]

Appendix II: Surgical DRGs identified by >10% of patients receiving blood or blood constituent transfusions. DRGs were combined when they involved similar surgery with similar bleeding frequencies (differences <3%).

| **DRG code** | **Name of DRG** | **Total Patients** | **No. WB** | **WB %** | **Grouped DRGs** |
| --- | --- | --- | --- | --- | --- |
| 05C02 | Valvular replacement with cardiopulmonary bypass with coronarography | 4382 | 803 | 18.30% | Valvular replacement |
| 05C03 | Valvular replacement with cardiopulmonary bypass without coronarography | 13575 | 2646 | 19.50% |
| 05C04 | Coronary bypass with coronarography | 4422 | 743 | 16.80% | Coronary bypass |
| 05C05 | Coronary bypass without coronarography | 11028 | 1609 | 14.60% |
| 05C06 | Pulmonary and cardiac surgery with cardiopulmonary bypass | 4906 | 692 | 14.10% | Other cardiac and vascular surgery |
| 05C08 | Pulmonary and cardiac surgery without cardiopulmonary bypass | 2849 | 321 | 11.30% |
| 05C10 | Major surgery for revascularisation | 31494 | 3708 | 11.80% |
| 05C11 | Other vascular surgery | 13115 | 1745 | 13.30% |
| 06C03 | Rectal resection | 18872 | 1953 | 10.30% | Rectal resection |
| 06C16 | Upper digestive tract surgery for cancer | 4958 | 694 | 14.00% | Upper digestive tract surgery |
| 06C20 | Oesophagus and gastric surgery for ulcus | 2623 | 387 | 14.80% |
| 06C21 | Digestive tract surgery by laparotomy | 5088 | 546 | 10.70% | Laparotomy and liver surgery |
| 07C09 | Liver, pancreas surgery for cancer | 6649 | 823 | 12.40% |
| 08C02 | Major hip and/or knee multiple surgery | 1302 | 322 | 24.70% | Revision and multiple orthopaedic surgery |
| 08C22 | Articular prosthesis re-operation | 22530 | 4153 | 18.40% |
| 08C24 | Knee prosthesis | 67324 | 7124 | 10.60% | Other orthopaedic surgery |
| 08C47 | Hip prosthesis for fracture | 32811 | 4191 | 12.80% |
| 08C49 | Hip and femur traumas age over 17 | 52509 | 6900 | 13.10% |
| 11C02 | Kidney or major bladder surgery for cancer | 15051 | 1800 | 12.00% | Kidney and spleen surgery |
| 16C02 | Spleen surgery | 1428 | 199 | 13.90% |
| 27C02 | Liver transplants | 953 | 173 | 18.20% | Transplantations |
| 27C03 | Pancreas transplants | 34 | 7 | 20.60% |
| 27C04 | Pulmonary transplants | 217 | 52 | 24.00% |
| 27C05 | Heart transplants | 349 | 88 | 25.20% |
